# Interrupted time series analysis of the impact of DIP reform on hospitalization costs in different types of hospitals

**DOI:** 10.3389/fpubh.2025.1644476

**Published:** 2025-10-16

**Authors:** Yan Chun-Hong, Lin Ke-Xin, Zheng Xin-Yue, Meng Xue-Hui

**Affiliations:** School of Humanities and Management, Zhejiang Chinese Medical University, Hangzhou, China

**Keywords:** Diagnosis-Intervention Packet (DIP), hospitalization costs, traditional Chinese medicine hospitals, general hospitals, interrupted time series analysis

## Abstract

**Objective:**

In the context of medical insurance payment reform, this study aims to evaluate the impact of the Diagnosis-Intervention Packet (DIP) payment policy on hospitalization costs across different types and levels of hospitals. In order to provide empirical evidence to support the high-quality collaboration between hospitals and medical insurance, while reducing the economic burden on patients.

**Method:**

Our study collected medical insurance reimbursement data from January 2019 to December 2022 in S city, covering 2,467,746 patients. Based on the intervention time point of the DIP reform implementation in 2021, an interrupted time series analysis was conducted on a monthly basis to compare the trend changes in hospitalization costs between traditional Chinese medicine hospitals (TCMHs) and general hospitals (GHs), as well as to examine the differences in impacts across hospitals of various levels.

**Results:**

Firstly, our study found that tertiary hospitals had the highest average hospitalization costs ( $Y_{\text{TCMH}3}=6170.33,Y_{\mathrm{GH}3}=12181.32$ ), followed by secondary hospitals ( $Y_{\text{TCMH}2}=4617.47,Y_{\mathrm{GH}2}=5344.60$ ), and primary hospitals, which had the lowest costs ( $Y_{\text{TCMH}1}=2490.93,Y_{\mathrm{GH}1}=1916.57$ ). Secondly, after the implementation of the DIP reform, the average hospitalization costs immediately decreased in both TCMHs and GHs, with a more significant reduction observed in GHs ( $\beta_{2-\text{TCMH}}$ = − 0.023, *p* = 0.059, $\beta_{2-\mathrm{GH}}$ = − 0.016, *p* = 0.039). Thirdly, when further categorized by hospital level, we found that the instantaneous effect of the reform on average hospitalization costs was most significant in primary TCMHs ( $\beta_{2-\text{TCMH}1}$ = − 0.080, *p* = 0.008), followed by tertiary TCMHs ( $\beta_{2-\text{TCMH}3}$ = − 0.033, *p* = 0.012), while the effect in secondary TCMHs was not significant ( $\beta_{2-\text{TCMH}2}$ = − 0.024, *p* = 0.087). In GHs, the most significant instantaneous effect was observed in tertiary hospitals ( $\beta_{2-\mathrm{GH}3}$ = − 0.046, *p* = 0.004), while no significant changes were observed in secondary and primary hospitals ( $\beta_{2-\mathrm{GH}2}$ = − 0.026, *p* = 0.077, $\beta_{2-\mathrm{GH}1}$ = − 0.022, *p* = 0.201). In terms of the long-term effects of the DIP reform, both TCMHs and GHs showed significant changes in average hospitalization costs, with a larger reduction observed in GHs, indicating better reform outcomes ( $\beta_{3-\text{TCMH}}$ = − 0.006, *p* < 0.001, $\beta_{3-\mathrm{GH}}$ = − 0.010, *p* < 0.001).

**Conclusion:**

The government should adjust policies in a differentiated and refined manner based on the type and level of hospitals to achieve the goals of controlling medical costs and improving the incentive mechanisms. Meanwhile, optimizing the healthcare service structure can improve quality and efficiency, as well as better meet patient needs.

## 1 Introduction

With changes in population structure, the transformation of disease patterns, and advancements in social technologies, the medical field has gradually faced pressing issues such as rapidly rising costs, deteriorating doctor-patient relationships, and increasing health inequalities. Among these, the rapid escalation of medical costs is particularly prominent, reflecting the growing disease burden on patients (1). Medical insurance, as an indispensable component of the whole healthcare system, the payment model it adopts plays a crucial role in the rational control of medical costs and the improvement of service quality (1, 2). In order to curb the rapid growth of healthcare costs, many countries have implemented reforms in insurance payment systems, among which the Diagnosis-Related Group (DRG) payment model has become the mainstream method for inpatient services, effectively limiting the overuse of medical resources. However, as the DRG reform progress, issues such as patient classification, cost shifting, and medical inequities have gradually emerged, particularly in resource-constrained, low- and middle-income countries (3, 4). As one of the earliest developing countries to adopt DRG payment, China has also faced these challenges. To explore a payment model that is easy to implement, rapidly scalable, and suited to the national context, the Chinese government officially launched and promoted its innovative insurance payment model—Diagnosis-Intervention Packet (DIP) in 2021 (3). DIP payment method, combines the total budget of the regional point method with the disease-based value-added payment, objectively classifying cases based on the logic of “disease diagnosis + treatment method,” and standardizing all case data within a given region (5, 6).

Compared to the DRG model, which establishes unified rules based on medical knowledge and clinical pathways, the DIP model, based on vast amounts of real clinical data and natural groupings formed through cluster analysis, can better accommodate the differences in diagnostic and treatment levels across regions and hospitals of varying tiers. It allows for more refined and flexible disease classification, covering a broader range of healthcare service scenarios (7). Furthermore, the DIP reform links hospital revenues directly to the regional budget and the hospital’s relative efficiency through “score values” and “point values,” introducing a competitive mechanism for horizontal comparisons between hospitals, thus further motivating hospitals to improve quality and efficiency. Therefore, as a major policy innovation originating from China’s practices, the DIP’s big data-driven and highly adaptable characteristics address many challenges faced by DRG during its initial implementation. However, its novelty and complexity also introduce new challenges. Consequently, conducting systematic and scientific research on the DIP reform to evaluate its effectiveness, mitigate risks, and optimize mechanisms is crucial for ensuring the success of healthcare payment reforms and guiding the high-quality development of the healthcare system.

Nowadays, the increasing burden of medical insurance expenditures has become a global concern. One of the central objectives of the reform of medical insurance payment methods is to control the unreasonable rise in medical costs. Hospitalization costs, as a key indicator of concern for hospitals, society, and government in the field of medical insurance reform, can directly reflect the effectiveness of policy cost control and reveal the behavioral strategies employed by hospitals to achieve financial surpluses. Moreover, a review of the literature shows that most studies on the effects of medical insurance reforms have incorporated hospitalization costs as a key metric. Therefore, this study has selected the average hospitalization cost as an indicator of the effects of DIP reform.

At present, the DIP reform is still in its early stages of policy implementation, and there is no consensus in the academic community regarding its effects (8). Some studies suggest that the DIP reform has been effective in controlling hospitalization costs and reducing the disease burden on patients, making it highly suitable for the Chinese healthcare environment (9, 10). Conversely, other studies point out that the implementation effects of DIP remain unstable (8). After categorizing hospitals based on the healthcare system, it was found that there are significant differences in the responses of TCMHs and GHs to the reform of medical insurance payment methods. The average hospitalization costs in TCMHs showed a clear upward trend post-reform, while those in GHs significantly decreased (11). Currently, the National Healthcare Security Administration of China have not issued a unified policy regarding medical insurance payments for TCMHs. As a result, the regulatory effect of medical insurance reforms on hospitalization costs in TCMHs is often not apparent, and TCMHs are relatively disadvantaged compared to GHs (12). This highlights the necessity of conducting research on the effects of DIP reform after classifying hospitals by type. Furthermore, these hospitals are classified into primary, secondary, and tertiary hospitals based on their technical capabilities, equipment levels, and the range of services they provide (13). Research on their performance under the DIP reform can not only reveal the differences in the adaptability and sensitivity of different types and levels of hospitals in resource allocation and medical services quality, but also provide valuable insights for evaluating and improving medical insurance payment systems, thereby promoting the overall efficiency and equity of healthcare services.

As one of the 71 pilot cities for DIP nationwide, City S has fully implemented this policy since 2021. The goal is to integrate the resources of all local medical institutions, form “a community with a shared future” among them, and enhance both cost control and service quality (14). The reasons for collecting samples from this region in our study are as follows. (1) Medical Resource Distribution: City S is at the medium level of socio-economic development in China, with nationally recognized top-tier hospitals and numerous first- and second-tier hospitals. The large sample size makes it highly representative. (2) Population Structure: As a typical industrial base and inland immigration city, City S has a balanced proportion of employees covered by employee insurance and urban–rural health insurance, as well as a similar ratio of local and cross-region patients. This makes it an excellent sample for studying the adaptability of medical insurance policies. (3) Policy Implementation: City S, recognized by the National Healthcare Security Administration of China as an exemplary reform pilot city, provides an advanced and representative setting for conducting research.

Interrupted Time Series (ITS) analysis, as an effective tool for accurately evaluating the actual impact of policies, has gained widespread attention from scholars both domestically and internationally (15–17). It is regarded as the most robust and commonly used quasi-experimental design for assessing the longitudinal effects of interventions (18). Meanwhile, with the continuous development of health policy, the application of ITS to evaluate health policies has become increasingly widespread in China (19). This method can objectively assess policy effectiveness by monitoring the trends and levels changes of indicators before and after policy implementation. Moreover, ITS is particularly well-suited for analyzing data in healthcare policy research, as it can effectively isolate potential confounding factors, ensuring the accuracy and reliability of the results. In the specific context of S city, ITS analysis can help clarify the impact of the DIP reform on hospitals of various types and levels, providing a scientific basis for policy evaluation and future policy development.

In conclusion, our study, based on data from 2,467,746 Inpatient samples in the exemplary pilot city S under the medical insurance payment reform, aims to use the ITS causal inference method to analyze and compare the changes in average hospitalization costs in TCMHs and GHs before and after the implementation of the DIP reform. Additionally, it further investigates the differences in responses to the DIP reform across hospitals of varying levels. The findings will provide empirical insights for policymakers, helping to reveal and optimize the implementation effects of the DIP reform in different types and levels of hospitals, thereby advancing the equity and efficiency of healthcare services.

## 2 Methods

### 2.1 Data resource

Our study collected all medical insurance reimbursement data for the period from January 2019 to December 2022 in S city. The data were obtained with approval from the city’s healthcare security administration and sourced from the city’s health insurance information platform. It covers information on 3,241,233 patients from 6 tertiary hospitals, 44 secondary hospitals, and 214 primary hospitals, totaling 248 GHs and 16 TCMHs. Each record was anonymized, and each patient was assigned a unique claim number, ensuring both the privacy and traceability of the data. The collected data encompass key information such as patient length of stay, hospitalization costs, hospital type, and hospital level. During the data cleaning phase, to ensure the reliability and validity of the results, we excluded cases that did not meet the DIP payment standards (those related to mental illness or rehabilitation), lacked essential patient information (such as hospitalization type, level, gender, or age), had missing or non-positive hospitalization duration or total costs, or had identical hospitalization numbers and discharge dates. Additionally, cases with abnormal hospitalization costs and durations were further removed using the Z-score method. In total, data from 2,467,746 patients were included in the study, with 271,550 from TCMHs and 2,196,196 from GHs.

### 2.2 Research method

In this study, hospitals in S city are classified into TCMHs and GHs, based on the main types of medical services. The two types differ significantly in service models, treatment approaches, and allocation of medical resources. TCMHs primarily utilize traditional Chinese diagnostic and therapeutic techniques, while GHs integrate both modern Western medicine and TCM, offering a wider range of medical services and technologies (15). The classification criteria are defined by national and local health departments, according to the regulations on medical institution management and service scope. Furthermore, hospitals are categorized into primary, secondary, and tertiary levels based on their service capabilities and facilities. The study period is divided into pre-reform (January 2019 to December 2020) and post-reform (January 2021 to December 2022), with the implementation of the DIP reform in January 2021 as the dividing line. The objectives of this study are to compare the changes in hospitalization costs before and after the DIP reform implementation in TCM and general hospitals, as well as to conduct a horizontal comparison across hospital levels, revealing the specific impact of the DIP reform on cost control in different types and levels of hospitals.

### 2.3 Statistical analysis methods

Our study uses ITS analysis to evaluate the effects of the DIP reform. We perform a logarithmic transformation processing on the hospitalization cost data with a skewed distribution. And standardize it based on China’s annual Consumer Price Index (CPI) to eliminate the impact of inflation (20). To assess the different performance between TCMHs and GHs in S city before and after the DIP reform implementation, our study firstly analyze the annual changes in total patient numbers, average length of stay, and average hospitalization costs. Then, using January 2021 as the key intervention point, we applied ITS method to examine the policy’s impact on average hospitalization costs.

The specific model is as follows:


Yt=β0+β1Tt+β2Xt+β3TtXt+εt

In the model, *Y_t_* represents the average hospitalization cost for each patient, and its logarithm was taken and included in the model for this study; *β_0_* is the estimated baseline level at the study’s start; *β_1_* indicates the trend slope prior to the DIP reform intervention; *β_2_* measures the immediate effect of the policy implementation; *β_3_* reflects the change in trend following the policy implementation; Tt is the time variable spanning the study period; *X_t_* is a dummy variable, taking the value 0 before the intervention and 1 after; *T_t_X_t_* represents the interaction between time and the policy intervention; *ε_t_* is the random error term.

Statistical analysis is conducted using Stata 16.0. To assess whether there is a serial correlation problem in the model, we conducted thorough diagnostic tests. First, the Durbin-Watson (DW) statistic was 1.85, close to 2, indicating no significant first-order autocorrelation. To further rule out higher-order serial correlation, we performed the Breusch-Godfrey LM test (lagged 3 periods), which yielded an LM statistic of 15.0 with a *p*-value of 0.002, suggesting the presence of some higher-order serial correlation. Therefore, to appropriately address the identified higher-order serial correlation and account for potential heteroscedasticity, we adopted the Newey-West HAC standard errors for statistical inference, ensuring the robustness of our results.

## 3 Results

### 3.1 The annual changes of inpatients in TCMHs and GHs

From 2019 to 2022, the total number of inpatient visits in TCM and general hospitals in S City is approximately 2,467,746. Annual variations in total inpatient visits, average length of stay, and average hospitalization costs are shown in Table 1.

**Table 1:** Annual situation of inpatients in TCMHs and GHs.

Variant	Traditional Chinese medicine hospitals			General hospitals		
	Total inpatient visits (person)	Average length of stay (day)	Average hospitalization costs (yuan)	Total inpatient visits (person)	Average length of stay (day)	Average hospitalization costs (yuan)
Tertiary hospitals						
2019	23051	11.41	6079.55	163092	9.09	12048.30
2020	13136	9.22	6248.91	112361	8.13	12622.12
2021	17115	9.98	6484.42	139674	8.18	12855.90
2022	14756	8.51	5868.43	128643	7.06	11198.96
On average	17014	9.78	6170.33	135942	8.12	12181.32
Secondary hospitals						
2019	65181	9.77	4337.65	222971	8.89	5088.60
2020	41497	8.82	4741.77	155919	8.08	5607.32
2021	44217	8.92	4872.33	182838	8.20	5665.86
2022	43241	7.99	4518.13	176209	7.32	5016.61
On average	48534	8.87	4617.47	184484	8.12	5344.60
Primary hospitals						
2019	2063	9.52	2855.18	261488	7.64	1746.52
2020	2253	8.11	2535.58	207125	7.38	2063.70
2021	2668	7.85	2299.76	229866	7.23	1987.69
2022	2372	7.33	2273.22	216010	6.90	1868.36
On average	2339	8.20	2490.93	228622	7.29	1916.57

A comparative analysis between TCM and general hospitals reveals that the total number of inpatient visits in general hospitals (GHs) is significantly higher than in TCM hospitals (TCMHs). In tertiary hospitals, the total number of inpatient visits in GHs is about eight times higher than in TCMHs. In secondary hospitals, this difference is approximately four times, and in primary hospitals, it is about 97 times. When comparing the average length of stay, TCMHs show slightly higher values than GHs, with the largest difference observed in tertiary hospitals, where the gap is 1.66 days. Regarding average hospitalization costs, secondary and tertiary GHs incur higher costs than TCMHs, particularly in tertiary hospitals, where the costs in GHs are nearly twice as high as those in TCMHs. However, in primary hospitals, the average cost in TCMHs exceeds that of GHs.

Analyzing the overall hospital levels, it can be seen that both the average length of stay and average hospitalization costs are highest in tertiary hospitals, followed by secondary hospitals, and lowest in primary hospitals. In terms of total patient visits, while TCMHs maintain this pattern, GHs show a different trend, with secondary hospitals have the highest average total visits, averaging 184,484 annually, followed by tertiary hospitals, and then primary hospitals.

Yearly trends indicate that in tertiary hospitals, both TCM and general hospitals exhibit fluctuations in total visits, average length of stay, and average hospitalization costs, with a noticeable decline in these metrics after 2021. In secondary hospitals, both TCM and general hospitals show fluctuating in total visits, with average hospitalization costs basically increasing prior to the DIP reform and decreasing afterward. In primary hospitals, the length of stay and hospitalization costs for TCMHs have consistently decreased throughout the study period.

### 3.2 Analysis results of ITS

#### 3.2.1 Analysis of the average hospitalization costs in TCMHs and GHs

**Figure 1:**
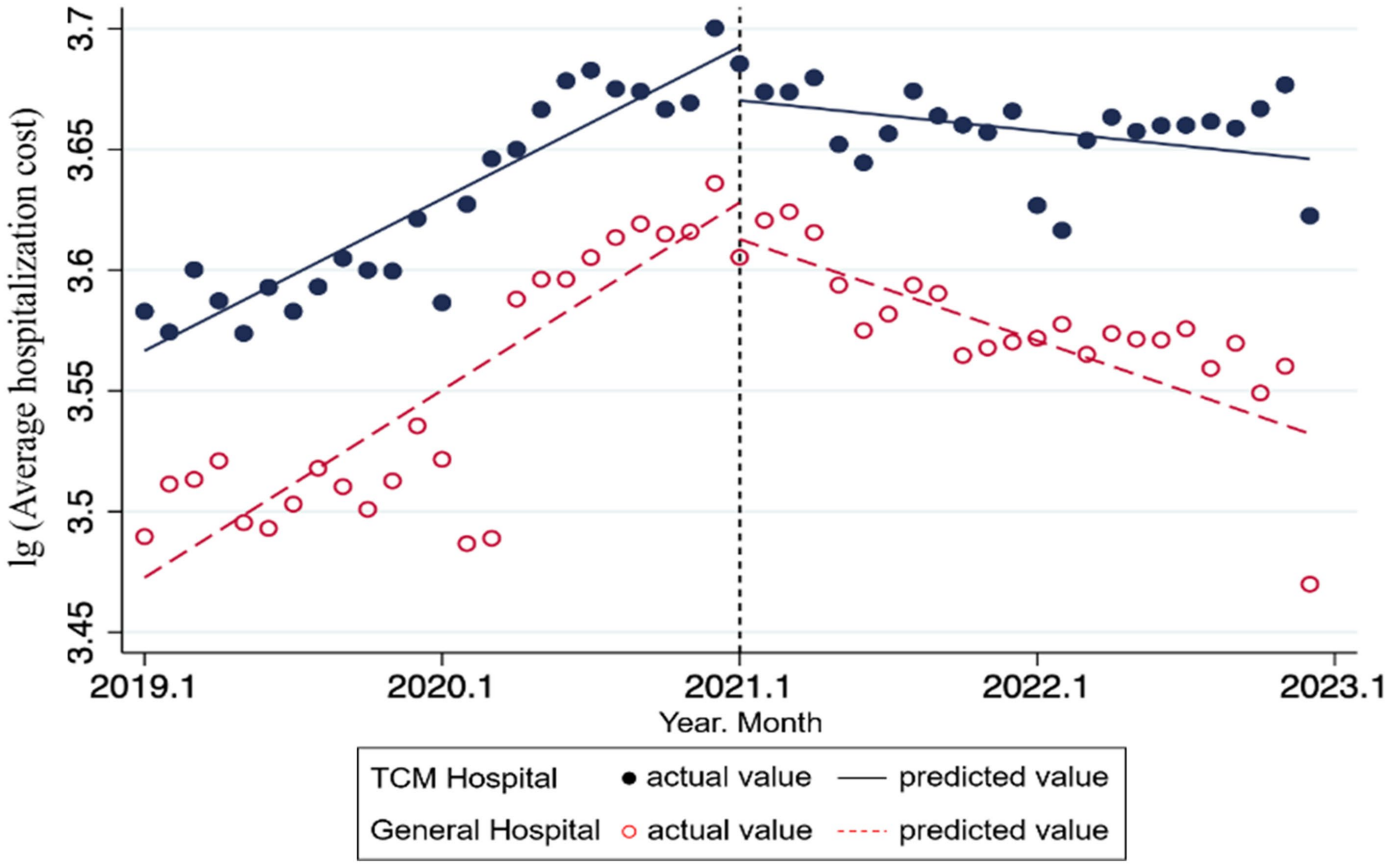
ITS trend of the average hospitalization costs in TCMH and GH.

In the result of ITS analysis presented in Table 2 and Figure 1, we observe that the DIP reform can significantly impact inpatient expenses in both TCMHs and GHs, with each month serving as a time unit. Specifically, prior to the reform, the average hospitalization costs in TCMHs increased significantly by 0.005 units per month $\left(\beta_{1-\text{TCMH}}=0.005,\;p<0.001\right)$ . Following the reform, the trend slope of average hospitalization costs decreased by 0.006 units per month $\left(\beta_{3-\text{TCMH}}=-0.006,\;p<0.001\right)$ , resulting in a post-reform slope of −0.001 units per month. In contrast, for GHs, prior to DIP reform, the average hospitalization costs increased significantly by 0.007 units per month $\left(\beta_{1-\mathrm{GH}}=0.007,\;p<0.001\right)$ . At the immediate point of reform implementation, there was a significant immediate level change (reduction) of 0.016 units $\left(\beta_{2-\mathrm{GH}}=-0.016,\;p=0.039\right)$ . Following the reform, the change in the trend slope was −0.010 units per month $\left(\beta_{3-\mathrm{GH}}=-0.010,\;p<0.001\right)$ , resulting in a post-reform slope of −0.003 units per month. These results indicate that the DIP reform can effectively reduce the rate of increase in hospitalization costs in both TCM and general hospitals, with a more pronounced cost control effect in GHs compared to TCMHs, consistent with previous studies (21, 22). Moreover, significant immediate reductions in hospitalization costs are observed in both hospital types at the time of policy implementation, further confirming the direct impact of the DIP reform on controlling healthcare costs. The reduction in average hospitalization costs resulting from the DIP reform can help save insurance funds, alleviate the financial burden on patients, enhance hospitals’ cost control capabilities, and promote more efficient hospital operations.

**Table 2:** ITS analysis of the average hospitalization costs in TCMHs and GHs.

Hospitalization costs	The monthly growth trend before DIP		The instantaneous changes at the time of DIP		The change in the slope of the trend after DIP	
	*β_1_* (95%CI)	*p*	*β_2_* (95%CI)	*p*	*β_3_* (95%CI)	*p*
TCMHs	0.005 (0.004, 0.007)	<0.001	−0.023 (−0.047, −0.009)	0.059	−0.006 (−0.008, −0.004)	<0.001
GHs	0.007 (0.004, 0.009)	<0.001	−0.016 (−0.054, 0.021)	0.039	−0.010 (−0.013, −0.007)	<0.001

#### 3.2.2 Analysis of the average inpatient expenses in three-level hospitals within TCMHs and GHs

Tables 3, 4 present the changes in overall average hospitalization costs before and after the DIP reform, with hospitals classified by level.

**Table 3:** ITS analysis of the average hospitalization costs in various levels of TCMHs.

Hospitalization costs	The monthly growth trend before DIP		The instantaneous changes at the time of DIP		The change in the slope of the trend after DIP	
	*β_1_* (95%CI)	*p*	*β_2_* (95%CI)	*p*	*β_3_* (95%CI)	*p*
Tertiary hospitals	0.006 (0.004, 0.007)	<0.001	−0.033 (−0.058, −0.008)	0.012	−0.007 (−0.009, −0.006)	<0.001
secondary hospitals	0.005 (0.004, 0.007)	<0.001	−0.024 (−0.051, 0.004)	0.087	−0.007 (−0.009, −0.005)	<0.001
primary hospitals	−0.005 (−0.009, −0.002)	0.003	−0.080 (−0.138, −0.022)	0.008	0.007 (0.002, 0.012)	0.011

**Table 4:** ITS analysis of the average hospitalization costs in various levels of GHs.

Hospitalization costs	The monthly growth trend before DIP		The instantaneous changes at the time of DIP	The change in the slope of the trend after DIP		
	*β_1_* (95%CI)	*p*	*β_2_* (95%CI)	*p*	*β_3_* (95%CI)	*p*
Tertiary hospitals	0.006 (0.004, 0.008)	<0.001	−0.046 (−0.077, −0.016)	0.004	−0.009 (−0.011, −0.006)	<0.001
Secondary hospitals	0.006 (0.004, 0.008)	<0.001	−0.026 (−0.055, 0.003)	0.077	−0.010 (−0.012, −0.007)	<0.001
Primary hospitals	0.005 (0.002, 0.007)	<0.001	−0.022 (−0.057, 0.012)	0.201	−0.007 (−0.011, −0.004)	<0.001

The result shows that in tertiary hospitals, DIP reform has a stronger instantaneous and long-term effect on hospitalization costs control in GHs. Prior to the reform, the average hospitalization costs increased significantly by 0.006 units per month $\left(\beta_{1-\mathrm{GH}3}=0.006,\;p<0.001\right)$ . At the immediate point of reform implementation, there was a significant immediate level change (reduction) of 0.046 units $\left(\beta_{2-\mathrm{GH}3}=-0.046,\;p=0.004\right)$ . Following the reform, the change in the trend slope was −0.009 units per month $\left(\beta_{3-\mathrm{GH}3}=-0.009,\;p<0.001\right)$ , resulting in a post-reform slope of −0.003 units per month. In comparison, for tertiary TCM hospitals, the average hospitalization costs prior to the reform increased significantly by 0.006 units per month $\left(\beta_{1-\text{TCMH}3}=0.006,\;p<0.001\right)$ . At the immediate point of DIP implementation, there was a similar immediate reduction of 0.033 units in hospitalization costs $\left(\beta_{2-\text{TCMH}3}=-0.033,\;p=0.012\right)$ . Following the reform, the change in the trend slope was −0.007 units per month $\left(\beta_{3-\text{TCMH}3}=-0.007,\;p<0.001\right)$ , resulting in a post-reform slope of −0.001 units per month.

In secondary hospitals, both TCMHs and GHs exhibited a significant decrease in the trend slope of average hospitalization costs following the reform’s implementation $(\;\beta_{3-\text{TCMH}2}=-0.007,p<0.001;\beta_{3-\mathrm{GH}2}=-0.010,p<0.001).$ However, the immediate level change at the point of reform implementation was not statistically significant for either hospital type $(\;\beta_{2-\text{TCMH}2}=-0.024,p=0.087;\beta_{2-\mathrm{GH}2}=-0.026,p=0.077),$ indicating a more limited immediate impact of the policy on this level of hospitals.

For primary hospitals, the GHs showed no statistically significant immediate response at the point of reform implementation ( $\beta_{2-\mathrm{GH}1}=-0.022,\;p=0.201$ ). In contrast, TCMH exhibited both a significant immediate reduction in costs ( $\beta_{2-\text{TCMH}1}=-0.080,\;p=0.008$ ) and a noteworthy reversal in the cost trend following the reform ( $\beta_{1-\text{TCMH}1}=-0.005,\;p=0.003,\beta_{3-\text{TCMH}1}=0.007,\;p=0.011$ ).

**Figure 2:**
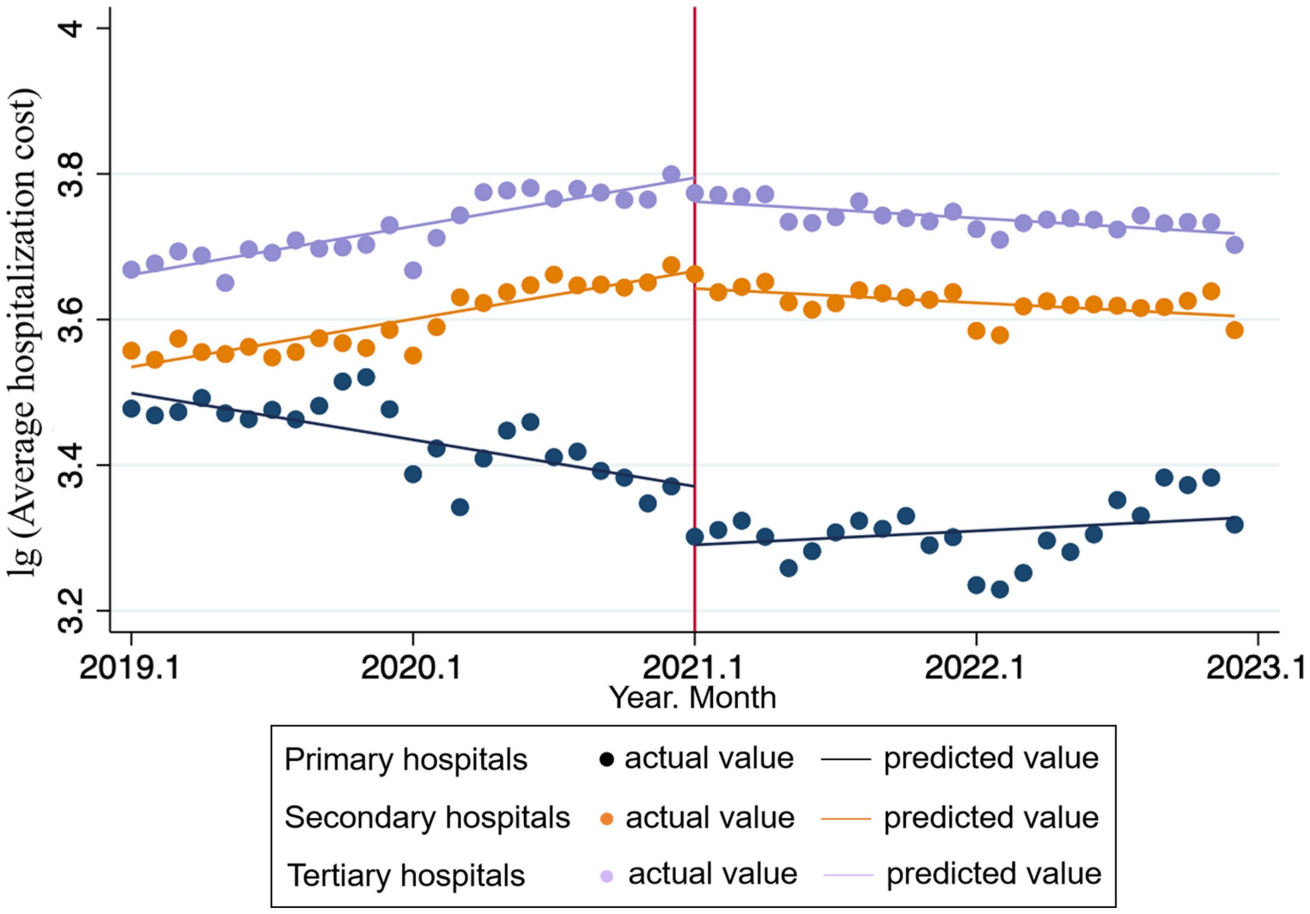
ITS trend of the average hospitalization costs in different levels of TCMHs.

**Figure 3:**
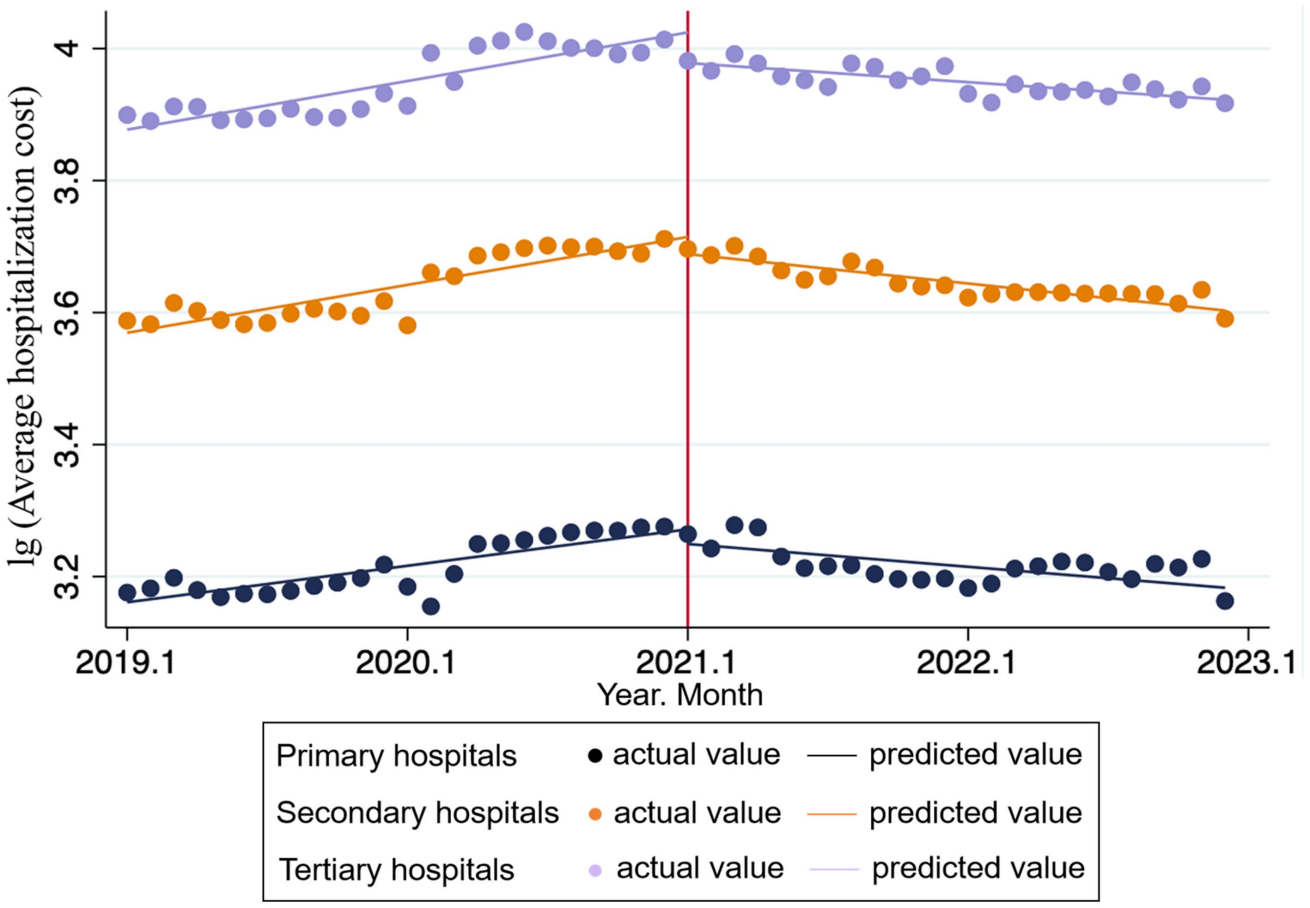
ITS trend of the average hospitalization costs in different levels of GHs.

While the DIP reform proves effective in slowing cost growth, particularly in resource-intensive tertiary GHs [consistent with prior studies (9, 23)], a more complex response pattern emerged in primary TCMHs. These hospitals exhibited a significant immediate cost reduction, followed by a subsequent rebound. This pattern can be attributed to a two-phase adaptation process. Initially, primary TCMHs readily complied with policy pressure by cutting non-core, adjustable services (e.g., high-value consumables and tests), resulting in short-term cost savings. However, in the longer term, structural constraints—such as the inherent rigidity of TCM cost structures, limited resource capacity, and the poor fit of the DIP grouping system for TCM practices—made it unsustainable to maintain strict cost control without compromising care quality or operational stability. Consequently, costs rebounded as hospitals prioritized therapeutic efficacy over aggressive cost containment. This finding highlights the unique challenges of applying DIP reforms to TCM, particularly in resource-limited primary settings. It underscores the necessity for future policies to move beyond a one-size-fits-all approach. To ensure sustainability, reforms must balance cost-control objectives with the preservation of TCM’s distinctive value through measures such as developing TCM-specific grouping methodologies and ensuring adequate reimbursement for core TCM services (see Figures 2, 3).

## 4 Discussion

### 4.1 Under the DIP reform, the cost response of TCMHs is slower than that of GHs

In the analysis of the impact of the DIP payment system reform on hospitalization costs in TCMHs and GHs, the study found significant and differing effects between the two types of hospitals. Both experienced a decline in average hospitalization costs immediately following the reform, with a more pronounced change in GHs ( $\beta_{2-\text{TCMH}}$ = − 0.023, *p* = 0.059, $\beta_{2-\mathrm{GH}}$ = − 0.016, *p* = 0.039). This difference may be attributed to several key factors, such as the hospitals’ service models, cost structures, resource allocation, technological development and its ability to adapt to policy changes (24–26). Firstly, GHs exhibit a more rapid cost adjustment in response to the DIP reform, indirectly reflecting their potential reliance on high-cost treatments and technologies. The Western medical system they encompass typically involves more costly high-tech medical equipment and complex surgical procedures (27). Under the direct regulation of the DIP reform, there is an immediate and significant adjustment in costs, specifically, it may be manifested as the active reduction of unnecessary high-priced consumables, the optimization of the diagnostic test combinations and so on. This adjustment not only reduces the cost of individual services but may also alter the overall strategy of hospital charging and service provision, driving a shift toward the standardization of clinical pathways and cost-intensive management. In contrast, although the cost response in TCMHs also shows a decline, the magnitude is smaller. In the term of disease perspective, Traditional Chinese Medicine primarily focuses on chronic disease treatment, emphasizing holistic and long-term adjustment (28). It aims to restore internal balance and health through harmonizing Yin and Yang and strengthening the body’s self-healing abilities. So that the therapeutic effects of TCM are generally slower than those of Western medicine. From the perspective of pharmaceuticals, the Chinese herbal medicines and TCM formulations used in TCMHs mostly come from natural sources, and their associated service and treatment costs involved are originally relatively fixed and low, making it difficult for the DIP reform to result in a rapid reduction in average hospitalization expenses. As a result, the immediate response to the DIP reform is less pronounced compared to GHs. Additionally, TCMHs rely less on expensive medical equipment, contributing to a more stable cost structure, thus reducing their sensitivity to the economic impacts of the DIP reform. These differing responses of TCMHs and GHs to the DIP reform not only reflect the economic structural differences between the two medical systems but also underscore the importance of considering medical diversity in policy implementation. Future healthcare policies should take into account the distinct characteristics of various medical systems and their influence on policy outcomes and hospital adaptation strategies. When adjusting medical insurance policies, policymakers need to conduct a more detailed analysis of hospital types and service models to ensure the fairness and effectiveness of the policies (e.g., by constructing a performance evaluation system that accommodates the principles of traditional Chinese medicine, ensuring sustainable compensation for long-cycle services such as disease prevention and management of chronic conditions), promote the rational allocation of medical resources, and maximize benefits for all patients (29).

### 4.2 Tertiary hospitals are the most sensitive to cost response under the DIP reform

Tertiary hospitals, as institutions providing advanced medical services, have responded quickly and significantly to the DIP reform, likely due to their reliance on high-cost medical activities in both financial and management structures (22). Consequently, any cost control measures will lead to substantial economic benefits. This phenomenon reflects that higher-level hospitals possess strong resource adjustment capabilities and policy adaptability. Their response mechanism essentially transforms payment pressure into a driving force for refined management, enabling effective optimization of service and cost structures under the new financial pressure. In contrast, secondary and primary hospitals exhibit milder responses, possibly due to their smaller scale, fewer resources, and less advanced medical technologies compared to tertiary hospitals (26). As a result, their adjustments following the policy implementation are less pronounced. Our study finds that the higher the hospital’s level, the greater its sensitivity to policy changes and ability to adjust, which may be attributed to the hospital’s financial resources, management structure, and the complexity of its medical services (30, 31). Moreover, although primary hospitals may not match higher-level hospitals in terms of economic scale and technical capabilities, they have demonstrated a reversal in hospitalization cost growth under the DIP reform. This inverse improvement may be closely related to the implementation of performance-based payment incentive mechanisms at the grassroots level and adaptive improvements in basic healthcare services (such as strengthening standardized management packages for chronic diseases and establishing community-hospital referral closed loops to reduce cost redundancies). This outcome highlights the unequal impact of policies across different hospital levels, suggesting that investment in primary medical services may positively effect on cost control. These findings are significant for policymakers. When formulating and adjusting medical insurance policies, it is crucial to consider the hospital’s level and service capacity to ensure effective policy implementation (15). Therefore, policy design should be more refined, taking into account differences in resource allocation, service delivery, and cost control across hospital levels, to promote both equity and efficiency within the medical system. This approach ensures that policies not only meet the actual demand for medical services but also enhance the overall responsiveness and adaptability of the entire medical system to policy changes.

### 4.3 The DIP reform should focus on the long-term cost control effects

The immediate and long-term cost control effects of the DIP reform show significant differences between TCMHs and GHs, which reflects the gradient of transmission efficiency of the prepayment system reform across different service systems. The immediate effect reflects the direct impact of the policy, manifested by a sudden decrease in hospitalization costs upon implementation of DIP. Particularly in high-level hospitals, such as tertiary hospitals, the costs in GHs drop significantly and immediately, and their adjustment mechanisms mainly rely on the reduction of highly elastic cost items, while TCMHs exhibit a similar trend. This immediate effect indicates the effectiveness and practicality of the DIP reform in controlling medical healthcare costs, this immediate effect demonstrates the effectiveness and practicality of the DIP reform in controlling healthcare costs, especially in constraining technology-intensive medical practices. The long-term effect reflects the sustained economic impact of the policy. In tertiary hospitals, after the policy’s implementation, the rate of hospitalization cost growth per time unit decreased by 0.009 (*p* < 0.001), suggesting the long-term cost control capability of the DIP reform. In contrast, the long-term effects in lower-level hospitals may be more moderate, but still show a deceleration in cost growth. The realization of these long-term effects depends on the ongoing execution of the policy and the hospital’s adaptive adjustments, which require comprehensive participation and support from the medical system. Overall, the DIP reform demonstrates effectiveness in both immediate and long-term cost control. However, the immediate effects are more easily observable, while the long-term effects require more time and sustained policy execution for validation. Policymakers should pay greater attention to achieving long-term effects (32) and continuously monitor the trends in hospital costs to ensure the policy’s enduring effectiveness. Furthermore, further research is needed to explore other factors influencing the DIP reform’s effectiveness, such as hospital management models, medical technology levels, and patient behavior (32–34), to enhance the policy’s specificity and applicability. Ultimately, the successful implementation of the policy requires close collaboration between the government, medical institutions, and patients to promote the sustainable development and optimization of the medical system.

### 4.4 Policy adjustment and the optimization of medical services

Policy adjustments should be tailored to the level and type of hospital. Our research indicates that hospitals of different levels and types respond differently to the DIP reform, with higher-level hospitals being more sensitive. Therefore, policymakers need to account for these variations to develop more effective measures.

Initially, policy adjustments should focus on optimizing the medical service structure to enhance both the quality and efficiency of medical services (35). In the context of the DIP reform, optimizing the medical service structure can be achieved by changing service delivery models, promoting clinical pathway management, and advancing medical technology (36–38). Redirecting more healthcare funding towards primary medical institutions and TCM institutions can further optimize resource allocation in primary healthcare, regulate unreasonable medical expenses, and continuously improve the quality of care. At the same time, policy adjustments and service structure optimization must consider the evolving needs of patients and the healthcare system (20). As social and economic development progresses and medical technology advances, increased patient health literacy and autonomy have driven the transformation of healthcare services towards a collaborative decision-making model. As patients’ expectations for medical services continue to rise, policy adjustments and service structure optimization should fully consider personalized patient needs and medical preferences (39). This includes strengthening the construction of primary healthcare services, improving basic medical security levels, enhancing communication with patients (e.g., establishing a unified feedback channel for healthcare reform, increasing publicity on healthcare knowledge), and establishing an integrated health information system (e.g., creating a unified national and provincial-level healthcare reimbursement platform, integrating it with hospital management information systems to support decision-making in healthcare payment reform) (40, 41). These efforts will improve the accessibility, convenience, and satisfaction of medical services, driving the optimization of the medical service structure and the rational control of medical expenses. Additionally, policy adjustments and medical service optimization require close collaboration between the government, medical institutions, and patients. The government should strengthen the supervision and evaluation of medical policies (42), and promptly adjust the policy measures to ensure the effective implementation and long-term effectiveness of the policies. For TCMHs, it is crucial to implement a healthcare payment system that aligns with the characteristics of TCM diagnosis and treatment as soon as possible. For instance, we can encourage pilot regions to explore a DIP-based TCM treatment grouping directory and attempt a payment reform that ensures equal reimbursement for TCM and Western medicine for the same conditions with equivalent outcomes. For primary hospitals, a performance fund for primary diagnosis and chronic disease management can be established, with a certain percentage drawn from the savings from the DIP global budget as a reward pool to incentivize primary healthcare institutions to provide high-quality services. This would also offer compensation for patients adhering to the primary diagnosis principle. Additionally, a DIP reform simulation platform could be developed to model the impact of different point-value adjustment schemes on hospital revenue, service behaviors, and patient costs. Machine learning methods could be used to test policies in a simulation environment, enhancing the scientific and predictive power of decision-making.

Medical institutions should actively respond to medical insurance policy adjustments, shifting from “passive cost control” to “active value creation.” A value-based healthcare performance system should be established, incorporating indicators related to health outcomes (e.g., functional improvement rates, quality of life scores), patient experience (e.g., satisfaction with the treatment process, doctor-patient communication), and healthcare reform efficiency (e.g., the proportion of diseases with costs below the regional average). The focus should be on true medical value, rather than purely economic benefits, creating a synergistic effect with the DIP reform through “incentive compatibility”.

Patients should enhance their participation and oversight, transitioning from “passive acceptance” to “active choice.” This includes cooperating with national policies, engaging in hospital-provided health education, and promoting optimized service supply through informed decision-making, collectively advancing the sustainability and optimization of the healthcare system.

## 5 Innovations and limitations

In terms of innovation, first, this study is based on the medical insurance settlement and case data of 2,467,746 patients from an entire city, covering a wide range and various types of cases. Second, we classify medical institutions into traditional Chinese medicine (TCM) hospitals and Western medicine hospitals, analyzing the heterogeneous effects of the DIP medical insurance payment reform on the control of average inpatient costs in TCM and general hospitals. Most existing literature on the effects of medical insurance reform focuses only on either TCM or Western medicine hospitals, with little research addressing both simultaneously. In terms of limitations, the DIP reform policy, an innovative initiative tailored to China’s national context, was pilot-tested in 2021. And the National Healthcare Security Administration of China was officially established in 2018, making the collection of insurance settlement data prior to the implementation of the DIP reform challenging. The data on hospital inpatient insurance settlements analyzed in our study were limited due to the difficulty in data collection, resulting in a relatively short time span. Additionally, since the database in S City lacks comprehensive variables, to ensure accuracy and representativeness, our study only consider the total hospitalization cost of patients as the dependent variable. Furthermore, the focus of our study is at the hospital level, without further exploration of the relationship between patient demographic information, disease type, and total hospitalization costs. Future research should address these challenges and limitations for optimization.

## Data Availability

The data analyzed in this study is subject to the following licenses/restrictions: The data contains private information of patients. The data for this study were sourced from the DRG database of S city. In compliance with local database management protocols, all data from the hospital were anonymized prior to release. The hospital has authorized the use of this anonymized database for research purposes, and at no point during the study were any personal details or patient identities accessed. Requests to access these datasets should be directed to MX-H, mengxuehui@aliyun.com.
